# Functional lateralization of lexical stress representation: a systematic review of patient data

**DOI:** 10.3389/fpsyg.2014.00317

**Published:** 2014-04-10

**Authors:** Katja Häuser, Frank Domahs

**Affiliations:** ^1^Department for Communication Sciences and Disorders, McGill UniversityMontreal, QC, Canada; ^2^Centre for Research on Brain, Language and Music, McGill UniversityMontreal, QC, Canada; ^3^Institute of Germanic Linguistics, Philipps-University MarburgMarburg, Germany

**Keywords:** word stress, representational knowledge, left hemisphere, right hemisphere, acquired disorders of language, dysprosody

## Abstract

According to the functional lateralization hypothesis (FLH) the lateralization of speech prosody depends both on its function (linguistic = left, emotional = right) and on the size of the units it operates on (small = left, large = right). In consequence, according to the FLH, lexical stress should be processed by the left (language-dominant) hemisphere, given its linguistic function and small unit size. We performed an exhaustive search for case studies of patients with acquired dysprosody due to unilateral brain damage. In contrast to previous reviews we only regarded dysprosody at the lexical level (excluding phrasal stress). Moreover, we focused on the representational stage of lexical stress processing, excluding more peripheral perceptual or motor deficits. Applying these criteria, we included nine studies reporting on 11 patients. All of these patients showed representational deficits in word stress processing following a lesion in their language-dominant hemisphere. In 9 out of 11 patients, it was the left hemisphere which was affected. This is a much more consistent pattern as found in previous reviews, in which less rigorous inclusion criteria may have blurred the pattern of results. We conclude that the representation of lexical stress crucially relies on the functioning of the language-dominant (mostly left) hemisphere.

## Introduction

According to the functional lateralization hypothesis (FLH; Van Lancker, 1980; Van Lancker Sidtis et al., 2006) the lateralization of speech prosody depends both on its function and the size of the linguistic unit it operates on. Processing of prosody with an emotional function is assumed to be accomplished by the right hemisphere, whereas prosody with a linguistic function should be processed by the left or language-dominant hemisphere. Moreover, the right hemisphere is assumed to operate on larger scale linguistic units such as phrases or sentences, while small units such as syllables should be processed by the left (language dominant) hemisphere. In consequence, according to the FLH, lexical stress should be processed by the left (language-dominant) hemisphere, given its linguistic function and small unit size (Van Lancker, 1980; Wong, 2002).

One relevant source of evidence for the FLH are neuropsychological case studies. If lexical stress processing is found to be impaired in subjects with unilateral brain damage, this would provide insights into the neural substrates that are necessarily involved in the processing of this aspect of prosody. However, so far such studies have yielded mixed results with respect to the FLH, and different reviews have arrived at conflicting results (Baum and Pell, 1999; Wong, 2002). Whereas the authors in one review concluded that there is sufficient evidence in favor of a consistent involvement of left hemisphere substrates in lexical stress processing (Baum and Pell, 1999), another review found the results too inconclusive to fully support the hypothesis of functional lateralization (Wong, 2002). These contradicting conclusions can partly be attributed to diverging methods and interpretations of the results. For example, Wong (2002) stated that since not all reviewed studies consistently include an LHD, RHD, and normal control group, some results are impossible to evaluate against the hypothesis of functional lateralization. Another potential problem is the fact that most previous studies have intermixed tasks involving different stages of lexical stress processing (such as perception, representation, and production), although it seems implausible that these processing stages are accomplished by the same neural regions at all (for a review, see Zatorre and Gandour, 2008). This has possible consequences for lateralization according to the FLH and could also explain why previous reviews did not reach a consistent conclusion in this matter. Finally, existing reviews often included clinical case studies conducted in English, some of which have insufficiently distinguished the size of the linguistic units under consideration. In some studies, compound noun phrases (green 'house vs. 'greenhouse) have been investigated on the same level as noun/verb minimal pairs ('convict vs. con'vict). Such an approach is potentially problematic, since noun phrases have greater semantic and syntactic complexity than compound nouns or simplex nouns and verbs (Wasow, 1997). Consequently, 'green house is not minimally distinct from green 'house in regards to word stress alone. Crucially, they also differ in the size of linguistic units involved which has implications for the lateralization of processing according to the FLH. In sum, various reasons ranging from differing methodologies to contrasting interpretations could explain the rather mixed evidence that has been discussed with respect to the FLH so far.

The goal of the present study was to review existing case reports with respect to the functional lateralization of lexical stress. In contrast to previous reviews (Baum and Pell, 1999; Wong, 2002), we only considered clinical case studies that investigated prosody at a purely lexical level, thus excluding studies on noun phrase or compound noun stress processing. Moreover, we focused on the representational stage of lexical stress processing, excluding perceptional and articulatory deficits. Our aim was to evaluate evidence informative to the claim that lexical stress is represented in the language-dominant (i.e., mostly left) hemisphere.

## Methods

We conducted an exhaustive search on the data bases Google Scholar, PubMed, and Entrez, using the search terms *lexical stress* AND *brain damage*, *lexical stress* AND *hemisphere*, and *lexical stress* AND *aphasia*. We discarded all studies that did not focus on individuals with unilateral brain damage and/or did not investigate stress assignment at a purely lexical level (for example, studies on tone or phrase level stress). In addition, we excluded all studies in which prosodic impairments in speech production could also result from more peripheral perceptual or articulatory difficulties (e.g., cases of dysarthric or apraxic impairment). After the application of these exclusion criteria, 12 articles remained for analysis, reporting on 15 patients with representational impairments in lexical stress processing (meaning that these patients displayed impairments in stress assignment). We reviewed all 12 studies for hemispheric site of lesion and language-dominant hemisphere of the patient. If both types of information were missing and could not be inferred based on the information provided by the authors, the study and/or subject was excluded from further analysis, resulting in the exclusion of four studies/subjects (excluded studies: Lloyd, 1999; Howard and Smith, 2002; Janssen, 2003. excluded subject: DE in Black and Byng, 1986). An overview on all studies and patients included is provided in Table 1.

**Table 1:** **Table of patients included in the review**.

**Study**	**Patient**	**Hand**	**Hemisphere**	**Site**	**Syndrome**	**Fluency**	**Etiology**	**Nam (%)**	**Read (%)**	**Repet (%)**	**Lex. Dec. (%)**
Chiacchio et al., 1993	CA	R	LH	T	Surface dyslexia, PPA	FL	DEG		30		
Galante et al., 2000	RM	R	LH	T, Ven	Surface dyslexia, PPA	FL	DEG		21		
Laganaro et al., 2002	MS	L + R	LH	x	Aphasia, Apraxia	NFL	CVA	10	12	10	7
Miceli and Caramazza, 1993	CLB	R	LH	T	Surface dyslexia	FL	CVA		26		
Rozzini et al., 1997	AC	R	LH	T, Ven	Surface dyslexia	FL	DEG		27		
Janssen and Domahs, 2008	HAT	R	LH, RH	T, Sub	Surface dyslexia, PPA	FL	DEG		25	2	
Marshall and Newcombe, 1973	ST	x	LH	T, P	Surface dyslexia	FL	OHI		xs		
	JC	x	LH	T, P	Surface dyslexia	FL	OHI		x		
Black and Byng, 1986	RW	x	LH	T, P	Deep dyslexia	FL	OHI		3		
	HRM	x	LH	x	Deep dyslexia, Agrammatism	NFL	CVA		3		
Coltheart et al., 1983	AB	L	RH	F	Surface dyslexia	x	OHI		x		
Cappa et al., 1997	GM	R	LH	T	Conduction aphasia	FL	OHI	18	14		

*Hand, reported handedness; Hemisphere, lesioned hemisphere; Site, site of lesion (if available); T, Temporal; Ven, Ventricle; P, Parietal; F, Frontal*.

*Etiology: DEG, Degenerative; OHI, Open Head Injury; CVA, Cerebral Vascular Accident*.

*Tasks: nam, picture naming; read, reading; repet, repetition; lex. dec., lexical decision task performance is indicated in % errors. x's indicate missing information*.

The languages spoken by the patients included in our analyses were English, German, and Italian—all languages with variable stress. This means that although in all three languages, word stress assignment shows some regularities (for an overview, see Van der Hulst, 1999), the assignment of stress to individual words cannot be inferred by phonemic or orthographic rules alone and thus requires activation of word-specific (i.e., lexical) phonological representations (Miceli and Caramazza, 1993). The word stress errors reported by the studies included in this review particularly affected words with infrequent or “irregular” stress patterns (Coltheart et al., 1983; Chiacchio et al., 1993; Miceli and Caramazza, 1993; Cappa et al., 1997; Rozzini et al., 1997; Galante et al., 2000; Laganaro et al., 2002; Janssen and Domahs, 2008), typically leading to shifts in stress assignment to the most frequent pattern (“over-regularisations”, e.g., Marshall and Newcombe, 1973; Black and Byng, 1986; Cappa et al., 1997; Laganaro et al., 2002; Janssen and Domahs, 2008).

## Results

### Classification based on handedness

This analysis is based on the fact that handedness is closely related to hemispheric dominance for language (e.g., Knecht et al., 2000). Out of the ten studies with 12 patients that remained in the pool (see Table 1), data on both the impaired hemisphere and the patient's handedness were available in eight cases. All of these eight patients presented with systematic errors in stress assignment following a lesion in their language-dominant hemisphere (which was the LH in seven patients and the RH in one patient), as inferred from handedness.

### Classification based on linguistic impairment

All 12 patients (including the four cases where handedness information was not available) showed major linguistic impairments, suggesting that they suffered from lesions in their language-dominant hemisphere. This yields a total of 12 out of 12 patients who showed representational deficits in word stress processing following a lesion in their language-dominant hemisphere. In 10 out of 12 patients, it was the left hemisphere which was affected.

## Discussion

The goal of this study was to review evidence from acquired language impairment regarding the functional lateralization hypothesis (FLH, Van Lancker, 1980; Van Lancker Sidtis et al., 2006), which states that function and size of a prosodic unit determine the cortical hemisphere it is processed in. Specifically, we were interested in the representation of lexical stress, which according to the FLH is a property of the left, language-dominant hemisphere. To this end, we reviewed clinical case studies that focused on brain-damaged patients with representational impairments in lexical stress assignment. Ten studies reporting on 12 cases remained for analysis after the application of all exclusion criteria. The results showed that in all of these patients, impairments in lexical stress assignment followed a lesion in the language-dominant hemisphere. In contrast to earlier reviews, which have arrived at mixed results, our data thus fully support the functional lateralization hypothesis.

The sample of studies that met our inclusion criteria was rather small, given that only few studies addressed the representation (rather than perception or articulation) of lexical stress. However, our rigorous and hypothesis-driven approach yielded a very clear pattern of results, in comparison to previous reviews that investigated speech prosody. In fact, a closer look at patients which were excluded from our analyses because they did not fulfill our criterion of a representational impairment (either because of a speech-motor deficit, e.g., dysarthria, or because of a non-specified deficit affecting lexical stress processing) revealed a much less consistent pattern: 82 of those patients had a left-hemisphere lesion, in comparison to 74 patients with right-hemisphere damage. Furthermore, 65 patients were reported to have lesions at the side of their dominant hand. Clearly, allowing for less precision in the nature of lexical dysprosody (as in previous reviews) would have led to a more impressive number of cases but to a blurred pattern of results. After all, it is highly plausible that perceptual and motor stages of lexical stress processing are subserved by bilateral brain areas whereas the more abstract linguistic representation of word prosody may reside in the language-dominant (left) hemisphere. This could explain the mixed evidence that earlier reviews yielded with respect to the FLH (Baum and Pell, 1999; Wong, 2002).

Our findings are consistent with evidence from dichotic listening showing that stress typicality effects (indicative of the representational stage of stress processing) only appeared in repetition and noun/verb-classification when stimuli were presented to the right ear/left hemisphere (Arciuli and Slowiaczek, 2007). More generally, our findings are also consistent with previous studies (Baum and Pell, 1999) that have rejected a strict division of labor regarding the hemispheric representation of prosody. Even though our results support the notion that lexical stress is a property of the language-dominant hemisphere, it seems that any global “all-left” or “all-right” account with respect to the hemispheric lateralization of *all* prosodic functions is an oversimplification and fails to account for the data. In this context, it seems that to date the FLH is the most promising account put forward to describe the neural substrates of prosody, since it does not set up an all-or-none division for prosodic functions but allows for gradedness of prosodic representation, depending on their function and the size of the processing units involved. This claim is also substantiated by findings in neuro-imaging, which have demonstrated bilateral cortical activations for lexical stress processing (Aleman et al., 2005; Wildgruber et al., 2006; Klein et al., 2011; Domahs et al., 2013). Yet, it is the methodological strength of lesion studies to highlight the functional relevance of brain regions for cognitive functions (Rorden and Karnath, 2004).

In sum, based on the data at hand we conclude that the representation of lexical stress crucially relies on the functioning of the language-dominant (mostly left) hemisphere.

### Conflict of interest statement

The authors declare that the research was conducted in the absence of any commercial or financial relationships that could be construed as a potential conflict of interest.
